# Longitudinal Antimicrobial Susceptibility Shifts in Environmental *Legionella pneumophila* from a Drinking Water Distribution System

**DOI:** 10.3390/pathogens15080852

**Published:** 2026-08-14

**Authors:** Lana Madagi, Maya Azrad, May Karp, Avi Peretz, Yehonatan Sharaby

**Affiliations:** 1 Department of Evolutionary and Environmental Biology, Faculty of Natural Sciences, University of Haifa, Haifa 3498838, Israel; lanahatuk93@gmail.com (L.M.);; 2 Clinical Microbiology Laboratory, Tzafon Medical Center, Poriya 1528001, Israel; 3 Azrieli Faculty of Medicine, Bar Ilan University, Safed 1311502, Israel; 4 Department of Biology and Environment, Oranim College, Kiryat Tivon 3600600, Israel

**Keywords:** *Legionella pneumophila*, Antimicrobial susceptibility, Drinking water distribution systems (DWDS), Environmental surveillance, Minimum inhibitory concentration (MIC)

## Abstract

*Legionella pneumophila* (*L. pneumophila*) persists in engineered water systems and is the causative agent of Legionnaires’ disease. Despite its clinical importance, little is known about whether environmental populations are undergoing gradual changes in their antimicrobial susceptibility. Here, we compared the antimicrobial susceptibility of 95 environmental *L. pneumophila* isolates collected in 2013–2014 with those obtained in 2024–2025 from the same sites in a drinking water distribution system (DWDS) using Epsilometer (E-test). The analyses showed both temporal and spatial variation: Ciprofloxacin activity remained relatively stable, whereas levofloxacin exhibited a broader minimum inhibitory concentration (MIC) range among contemporary isolates, with the minimum inhibitory concentration required to inhibit the growth of 90% of isolates (MIC_90_) doubling from 0.5 to 1.0 mg/L, suggesting increased variability and the emergence of less susceptible subpopulations. Tigecycline showed a marked site-specific increase in MIC values, with the mean MIC at site D increasing from 0.26 mg/L in the 2013–2014 isolates to 1.86 mg/L in the 2024–2025 isolates, indicating reduced susceptibility at this site. Among macrolides, erythromycin exhibited the lowest MIC values across both isolate collections, whereas azithromycin showed higher MIC values. Moreover, isolates recovered from adjacent sites within the same DWDS exhibited significantly different susceptibility profiles, possibly reflecting the influence of local environmental conditions. These findings emphasize that *L. pneumophila* susceptibility is not static but is likely shaped by ecological and temporal factors. The study underscores the importance of long-term environmental surveillance to track the evolution of this pathogen and provides new insights into how micro-scale dynamics within water systems can influence antimicrobial susceptibility patterns.

## 1. Introduction

*Legionella pneumophila* (*L. pneumophila*) is the causative agent of Legionnaires’ disease, a severe form of community-acquired pneumonia. Even with timely and appropriate antimicrobial therapy, reported mortality rates range between 5 and 15% in general populations and may rise substantially in severe or immunocompromised cases up to 40% in high-risk groups [1,2]. Moreover, the incidence of Legionnaires’ disease has increased steadily over the past decade. According to the European Centre for Disease Prevention and Control (ECDC), the notification rate in the EU/EEA reached 3.4 cases per 100,000 population in 2024, the highest level reported to date [3].

The bacterium colonizes both natural and engineered aquatic environments (e.g., lakes, rivers, drinking-water distribution systems (DWDS), hospital plumbing, and cooling towers) and commonly persists within biofilms and protozoan hosts, which confer protection from environmental stresses and facilitate transmission to humans via aerosolized water droplets [4,5,6]. Persistence is further promoted by water stagnation, temperatures between 25 and 42 °C, scale and sediment accumulation, and insufficient disinfectant residuals, all of which favor long-term persistence in engineered water systems [3,7]. Macrolides (e.g., azithromycin, clarithromycin) and fluoroquinolones (e.g., levofloxacin, ciprofloxacin) remain the mainstays of antimicrobial therapy for *L. pneumophila* infections [8]. Historically, *L. pneumophila* was considered broadly susceptible to first-line macrolides and fluoroquinolones, and antimicrobial resistance was not perceived as a major clinical concern. This perspective was reinforced by the fact that person-to-person transmission is considered exceptionally rare, with only a single probable case reported to date [9]. Consequently, the human host is generally regarded as an evolutionary dead end for the bacterium, as adaptations arising during infection are unlikely to become established in the environmental reservoir [2,10,11]. However, recent studies have challenged this assumption by demonstrating reduced antimicrobial susceptibility and the emergence of resistance-associated mechanisms in environmental *L. pneumophila* isolates [12,13,14].

A major challenge in antimicrobial susceptibility research is the lack of standardized susceptibility testing protocols, which limits comparisons across studies and over time. Michel et al. [15] and portal et al. [16] emphasized that methodological variability and inconsistent interpretive criteria impede comparability over time and between laboratories. While frameworks such as epidemiological cut-off values (ECOFFs) exist, their implementation remains inconsistent, and clinical breakpoints for *L. pneumophila* are lacking [4,14,17].

Moreover, surveillance data from drinking water in the Middle East is notably scarce. Most published reports originate from Europe, North America, or East Asia, creating a substantial regional information gap. Although *L. pneumophila* prevalence has been investigated in several regional studies in the Middle East [18,19,20,21,22,23], comprehensive studies of its antimicrobial susceptibility in this geographic region remain virtually non-existent except for Sharaby et al. [24].

In the study by Sharaby et al. [24], 105 environmental and clinical *L. pneumophila* isolates were genotyped using a multiple-locus variable-number tandem-repeat analysis of eight loci (MLVA-8) scheme and tested for susceptibility to ten antibiotics using the Epsilometer (E-test) methodology on buffered charcoal yeast extract (BCYE) agar. The authors reported generally intermediate minimum inhibitory concentration (MIC) values across most antibiotics tested, with no evidence of high-level resistance, although significant variations in susceptibility were observed between genotypes. These findings provided the first systematic antimicrobial susceptibility dataset for *L. pneumophila* in the region, but they were limited to a single time period, making temporal trend analysis impossible.

To address these gaps, the present study aimed to examine the temporal and spatial changes in the antimicrobial susceptibility of environmental *L. pneumophila* isolates obtained from the same sites within a single DWDS across two distinct time periods: a historical collection (2013–2014) and a contemporary collection (2024–2025). All isolates were analyzed using the same standardized methodology, ensuring rigorous methodological consistency and enabling direct comparison between the two sampling periods. This design enables robust temporal and spatial comparisons of *L. pneumophila* populations colonizing the same DWDS for the first time. Focusing on a minimally studied geographic region, this study offers novel, high-resolution insight into the potential evolution and stability of *L. pneumophila* antimicrobial susceptibility.

## 2. Materials and Methods

### 2.1. L. pneumophila Strains

**Figure 1:**
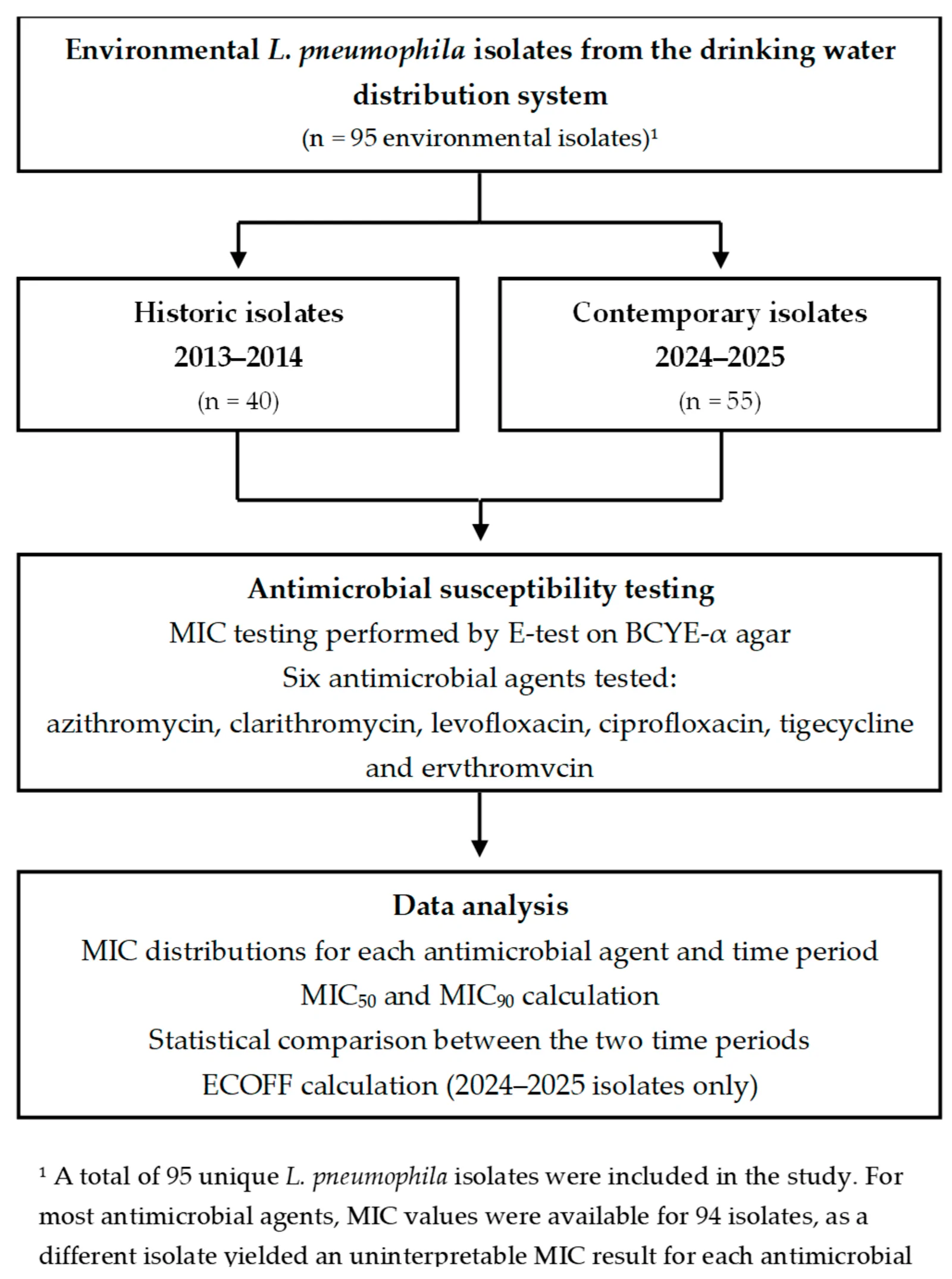
Study design, isolate selection, and antimicrobial susceptibility testing workflow.

We examined the susceptibility of 95 environmental isolates of *L. pneumophila* to six antimicrobial agents frequently employed for the treatment of legionellosis (Table 1, Figure 1). Among them, 40 were classified as “historic” isolates, obtained from a DWDS in northern Israel during a two-year study conducted between 2013 and 2014 (coordinates 32°42′43.17″ N, 35°6′28.66″ E). The sampling campaign covered six sampling sites (sites A–F), each representing a different building within the same DWDS, where seasonal monitoring was performed in both water and biofilm. *Legionella* isolation followed ISO 11731:1998 procedure, as detailed by Rodríguez-Martínez et al. [21].

The 55 “contemporary” isolates were collected from the same six sites (A–F) during 2024–2025, ensuring a contemporary dataset for direct temporal comparison. Importantly, the entire sampling and isolation process was performed exactly as in 2013–2014, using the same sampling protocol, culture conditions, and isolation procedures (ISO 11731:2017) [25]. Briefly, 1 L water samples were concentrated by filtration through 0.22 µm pore size membranes, which were subsequently resuspended in 5 mL sterile saline. Biofilm material was detached from pipe surfaces or fixtures by swabbing or scraping into sterile water. To reduce background microbiota, samples were subjected to heat pretreatment (50 °C, 30 min). Following heat pretreatment, 0.3 mL of each treated sample suspension was inoculated onto each BCYE agar plate supplemented with L-cysteine and iron. Plates were incubated at 37 °C for up to 10 days, and presumptive *Legionella* colonies were subcultured onto BCYE agar for five successive passages to obtain pure isolates, followed by species-level identification using standard microbiological and molecular methods.

### 2.2. Legionella Identification

Genomic DNA was extracted from all isolates using the ZymoBIOMICS DNA Miniprep Kit (Zymo Research, Irvine, CA, USA). Genus-level confirmation was performed by polymerase chain reaction (PCR)amplification using *Legionella*-specific primers Lgsp17F (5′-GGCCTACCAAGGCGACGATCG-3′) and Lgsp28R (5′-CACCGGAAATTCCACTACCCTCTC-3′) described by Pereira et al. [26], targeting a ~421 bp fragment of the 16S rRNA gene (V3–V4 region). Amplicons were resolved on 1.8% agarose gels prepared with RedSafe nucleic-acid stain (iNtRON Biotechnology, Seongnam-si, Korea) and electrophoresis was carried out for 30 min at 80 V. DNA from *Aeromonas veronii* and sterile nuclease-free water were included as negative controls, while DNA from *L. pneumophila* strain Philadelphia (ATCC 33152) served as the positive control alongside a molecular size ladder (Abert SuperLadder 2100, Abert Biotechnology, Taipei City, Taiwan). Only isolates displaying a clear amplicon of the expected size were retained for sequencing and subsequent analyses.

Species-level identification was primarily performed by PCR amplification and sequencing of the macrophage infectivity potentiator (*mip*) gene using primers described by Ratcliff et al. [27], which amplify a ~650 bp fragment. Amplicons were purified and subjected to Sanger sequencing at MCLAB (South San Francisco, CA, USA). Sequences were inspected, trimmed, and edited using Chromas version 2.6.6 (Technelysium Pty Ltd., South Brisbane, Australia) and high-quality consensus sequences were subsequently analyzed using the NCBI BLAST+ tool 2.17.0 [28]. Isolates showing ≥99% sequence identity to reference sequences were assigned at the species level. For isolates in which *mip*-based analysis did not yield an unambiguous species identification, PCR amplification and sequencing of the *dotA* gene were additionally performed using primers described by Ko et al. [29], yielding a ~420 bp fragment. Species assignments were confirmed when *dotA*-based BLAST results supported the *mip*-based assignment. Following species confirmation and purification, all *L. pneumophila* isolates were stored at −80 °C until further analyses.

### 2.3. Reference Strains

*L. pneumophila* Philadelphia (ATCC 33152) was used as the reference strain. In addition, *Staphylococcus aureus* (ATCC 29213) and *Escherichia coli* (ATCC 25922) were also selected for validation of susceptibility testing results (Table 1). The selected strains were kept frozen at −80 °C prior to analysis. The MIC values obtained for the quality-control strains *S. aureus* (ATCC 29213) and *E. coli* (ATCC 25922) were consistent with established reference ranges reported in the literature and by European Committee on Antimicrobial Susceptibility Testing (EUCAST) [30], confirming the reliability of the susceptibility testing procedure (Table 1). Although the MIC values obtained for *L. pneumophila* ATCC 33152 differed from those reported previously by Sharaby et al. [24], such variation has been documented for *Legionella* susceptibility testing and may reflect methodological differences associated with BCYE-based assays [16]. Importantly, all historical and contemporary isolates were analyzed using an identical protocol, allowing direct comparison within the present study.

### 2.4. Determination of MICs

The antimicrobial susceptibility of the isolates was determined following the same protocol previously described by Sharaby et al. [24]. Briefly, isolates were subcultured once on BCYE-α plates (BD Diagnostics Sparks, Sparks Glencoe, MD, USA) and suspended in 0.85% NaCl to a turbidity equivalent to 0.5 McFarland standard and tested using MIC gradient test strips (bioMérieux, Marcy-l’Étoile, France) on BCYE-α agar plates. Sterile cotton swabs were dipped in the inoculum suspension and evenly streaked across the entire agar surface. After allowing plates to dry for 10 min, E-test strips containing the relevant antimicrobial agent were applied. The following antimicrobials were tested: azithromycin, clarithromycin, levofloxacin, ciprofloxacin, tigecycline and erythromycin. Plates were incubated at 35 ± 1 °C in 2.5% CO_2_ for 48 h, and MIC values were recorded as the lowest antimicrobial concentration at which the inhibition ellipse intersected the test strip.

ECOFFs were calculated for the 2024–2025 isolates only using the EUCAST Gaussian-fit approach [31]. According to EUCAST, ECOFF is defined as the MIC value that encompasses ≥ 95% of the wild-type population [32].

### 2.5. Statistical Analysis

All statistical analyses were conducted in R version 4.4.0 [33] using a combination of packages from the tidyverse [34] for data processing and visualization, along with statistical packages including permuco [35], coin [36], and fitdistrplus [37]. Descriptive statistics, including means, standard deviations, medians, ranges, and modes were calculated for each antibiotic, stratified by isolate age and sampling site. To explore the overall variation in MIC distributions between antibiotics, a repeated-measures ANOVA was conducted across antibiotics within isolates. When the sphericity assumption was not met or data deviated from normality, MIC values were log_2_-transformed and reanalyzed. A significant main effect was followed by pairwise comparisons between antibiotics using paired Wilcoxon signed-rank tests with Holm correction.

Associations between MIC values of the six tested antibiotics were evaluated using Spearman’s rank correlation coefficients. Analyses were performed for the pooled dataset and repeated separately for historic and contemporary isolates to assess temporal differences in co-susceptibility patterns. Pairwise complete observations were used for each antibiotic pair. Two-sided *p*-values were calculated for all 15 pairwise comparisons within each correlation matrix and adjusted separately using the Benjamini–Hochberg false discovery rate (FDR) correction [38]. Correlations with FDR-adjusted *p*-values < 0.05 were considered statistically significant.

To examine the effect of sampling site and temporal origin (“historic” vs. “contemporary” isolates) on antibiotic susceptibility, permutation-based two-way analysis of covariance (ANCOVA) models were fitted for each antibiotic. The models included sampling site and age group as fixed factors, and MIC values as the dependent variable. Where necessary, MIC values were transformed using the base-2 logarithm. The models were estimated using the Freedman–Lane permutation method with 9999 permutations, implemented via the aovperm() function in the permuco package. This method controls covariates while testing effects of interest in a non-parametric framework, which is particularly robust for non-normally distributed microbiological data. Following significant interaction or main effects in the ANCOVA models, we conducted pairwise post-hoc comparisons using estimated marginal means (emmeans package) and adjusted *p*-values using the FDR method [38]. Additionally, simple-effects tests were performed within each sampling site to compare MICs between historic and contemporary isolates using the Wilcoxon rank-sum test (two-sided, unpaired). Holm correction was applied to adjust for multiple comparisons.

## 3. Results

**Figure 2:**
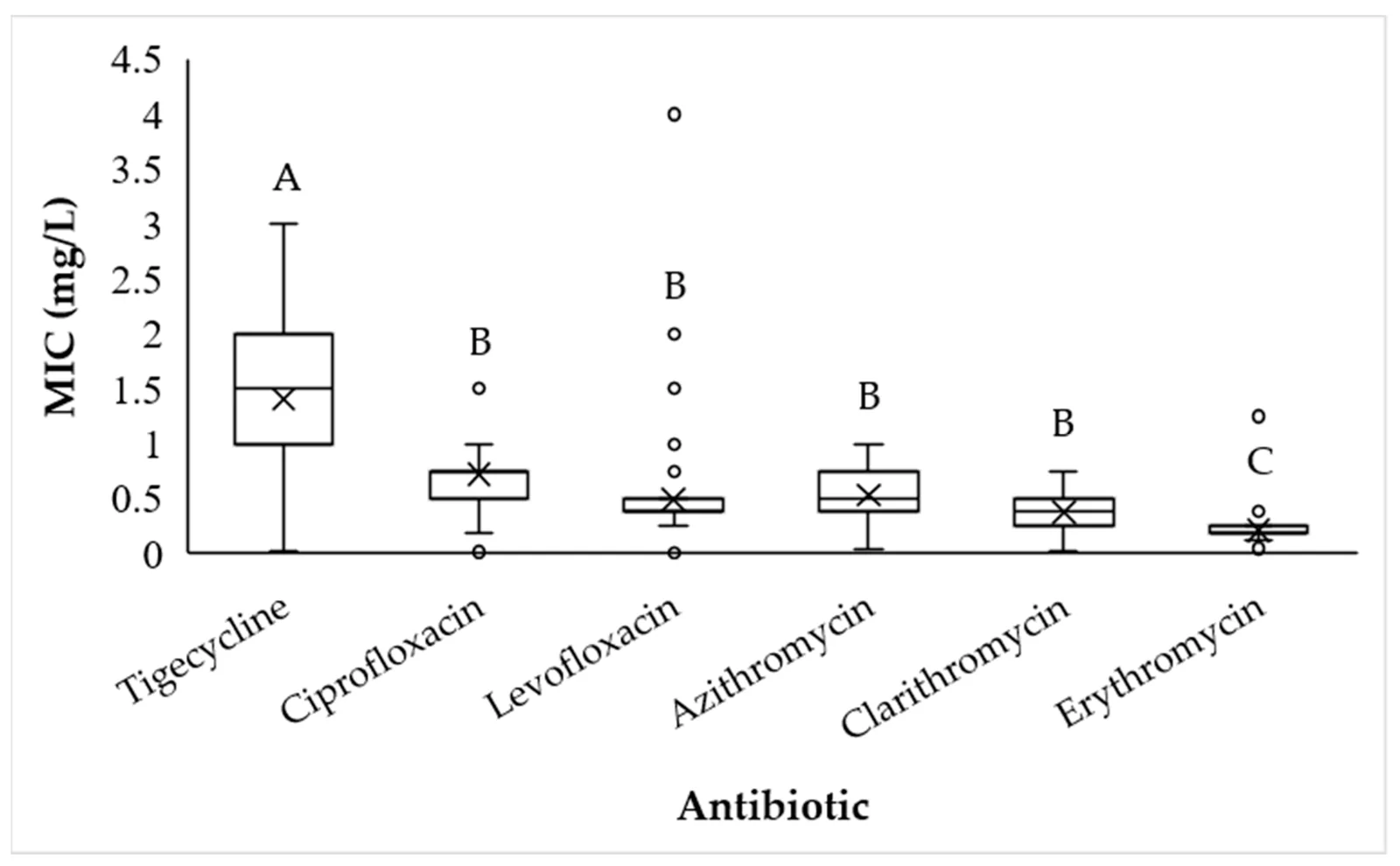
Boxplot distributions of minimum inhibitory concentrations (mg/L) for six antibiotics across all *L. pneumophila* isolates: azithromycin (*n* = 94), levofloxacin (*n* = 94), tigecycline (*n* = 94), ciprofloxacin (*n* = 93), clarithromycin (*n* = 93), and erythromycin (*n* = 94). Means are marked by X and circles indicate outlier values. Different letters above boxplots represent significant differences based on Tukey’s HSD post-hoc test following Holms’s correction for multiple comparisons.

MICs were determined for six antibiotics across 95 *L. pneumophila* isolates using E-test strips on BCYE agar. Repeated measures ANOVA revealed significant differences in antimicrobial susceptibilities across the six studied antibiotics (F_5,445_ = 92.73, *p* < 0.0001). Post-hoc comparisons using Tukey’s HSD test indicated that tigecycline exhibited the highest MIC values, whereas erythromycin showed the lowest MICs (*p* < 0.0001). Azithromycin, clarithromycin, ciprofloxacin and levofloxacin displayed intermediate MIC distributions, with no statistically significant differences among these agents (*p* > 0.05), as shown in Figure 2.

Tigecycline had the highest mean MIC at 1.41 ± 0.06 mg/L and a narrow interquartile range centered around a median and mode of 1.5 mg/L. MICs for ciprofloxacin ranged from 0.016 to 1.5 mg/L, with both the median and mode at 0.75 mg/L, indicating a compact distribution centered within the lower portion of the observed MIC range. Clarithromycin showed low MIC variability (mean 0.38 ± 0.01 mg/L), with values ranging between 0.016 and 0.75 mg/L and a predominant mode of 0.38 mg/L. Azithromycin exhibited a unimodal MIC distribution ranging from 0.047 to 1 mg/L, with a mean of 0.53 ± 0.03 mg/L, and both median and mode at 0.5 mg/L. Levofloxacin showed greater dispersion, with MICs spanning between 0.012 and 4 mg/L (mean 0.49 ± 0.05 mg/L, median 0.38 mg/L), consistent with a right-skewed distribution. Erythromycin exhibited the lowest MICs among all tested agents (mean 0.21 ± 0.01 mg/L, median and mode of 0.19 mg/L), with most isolates falling well below 0.5 mg/L. Overall, MIC distributions were narrow for macrolides and fluoroquinolones, whereas tigecycline showed evidence of elevated MICs in a subset of isolates.

Spearman correlation analysis was performed to evaluate associations among MIC values for the six tested antibiotics. *p*-values were adjusted for multiple comparisons using the FDR correction within each correlation matrix. The complete stratified correlation results are provided in Supplementary Table S2. In the pooled dataset, six of the 15 pairwise antibiotic associations remained significant after FDR correction. Azithromycin MICs were positively correlated with levofloxacin (r_s_ = 0.34, *p* = 0.0029), clarithromycin (r_s_ = 0.36, *p* = 0.0018), and erythromycin (r_s_ = 0.57, *p* < 0.001). Levofloxacin MICs were positively correlated with ciprofloxacin (r_s_ = 0.39, *p* < 0.001) and clarithromycin (r_s_ = 0.45, *p* < 0.001), while clarithromycin was also correlated with erythromycin (r_s_ = 0.25, *p* = 0.0357).

To determine whether these association patterns differed between sampling periods, the analysis was repeated separately for historic and contemporary isolates. Among historic isolates, significant positive correlations were observed for azithromycin–tigecycline (r_s_ = 0.36, *p* = 0.0456), azithromycin–clarithromycin (r_s_ = 0.64, *p* < 0.001), azithromycin–erythromycin (r_s_ = 0.58, *p* < 0.001), levofloxacin–clarithromycin (r_s_ = 0.44, *p* = 0.0179), levofloxacin–erythromycin (r_s_ = 0.41, *p* = 0.0257), tigecycline–erythromycin (r_s_ = 0.37, *p* = 0.0455), and clarithromycin–erythromycin (r_s_ = 0.49, *p* = 0.0071). In contrast, among contemporary isolates, only three associations remained significant after FDR correction: azithromycin–erythromycin (r_s_ = 0.51, *p* = 0.0014), levofloxacin–ciprofloxacin (r_s_ = 0.47, *p* = 0.0025), and levofloxacin–clarithromycin (r_s_ = 0.47, *p* = 0.0025).

These results indicate that co-susceptibility patterns were not uniformly strengthened over time. Rather, the structure of antibiotic MIC correlations differed between sampling periods: historic isolates showed stronger macrolide-associated correlations, whereas contemporary isolates showed a more restricted pattern dominated by correlations involving levofloxacin and the persistent azithromycin–erythromycin association.

### 3.1. Current State of Antibiotic Susceptibility

In the 2024–2025 dataset (contemporary collection), the observed MIC ranges and calculated ECOFF values varied markedly among the tested antibiotics, reflecting differences in MIC distributions. Ciprofloxacin displayed a relatively narrow MIC range (0.016–1.5 mg/L) with an ECOFF of 2.0 mg/L, consistent with the observed MIC distribution (Table 2).

ECOFFs were calculated with the EUCAST Gaussian-fit approach for the contemporary isolates (2024–2025). EUCAST defines ECOFF as the MIC that encompasses ≥ 95% of the wild-type population [32]. ECOFF values calculated in the current study were used solely to characterize the phenotypic wild-type MIC distributions and were not interpreted as clinical susceptibility breakpoints.

Levofloxacin showed a comparable minimum inhibitory concentration required to inhibit the growth of 50% of isolates (MIC_50_) of 0.38 mg/L but exhibited the broadest MIC range (0.012–4.0 mg/L). The calculated ECOFF of 1.5 mg/L was consistent with the observed MIC distribution of the contemporary isolates. Tigecycline demonstrated consistently higher MIC values compared to the fluoroquinolones, with a MIC_50_ of 1.5 mg/L, minimum inhibitory concentration required to inhibit the growth of 90% of isolates (MIC_90_) of 2.0 mg/L, and an ECOFF of 2.0 mg/L, consistent with the upper limit of the observed MIC distribution of the contemporary isolates. Among macrolides, azithromycin showed a moderate MIC range (0.047–1.0 mg/L) and an ECOFF of 1.0 mg/L, while clarithromycin had a lower MIC range (0.016–0.75 mg/L), accompanied by lower MIC_50_ and MIC_90_ values compared to azithromycin, while sharing the same ECOFF of 1.0 mg/L (Table 2). Erythromycin had the lowest ECOFF among all tested agents (0.25 mg/L) and a narrow MIC range (0.047–0.38 mg/L), reflecting limited variability in MIC values across the contemporary isolates. Taken together, these findings indicate that fluoroquinolones retain generally low MIC_50_ values but differ in the extent of MIC variability, whereas macrolides, particularly erythromycin, remain the most active agents against the recent isolate collection, and tigecycline appears to be the least potent.

### 3.2. Comparison Between Sampling Sites and Isolates Age Groups

To assess whether *L. pneumophila* strains isolated in earlier years (2013–2014, “historic”) differ in their antimicrobial susceptibility profiles compared with more recent isolates (2024–2025, “contemporary”), cumulative inhibition curves were compared for each antimicrobial agent as shown in Table 3. A comparison of the MIC_50_ and MIC_90_ values for the historical isolates with those previously reported by Sharaby et al. [24] is provided in Supplementary Table S1.

Overall, the contemporary isolates exhibited consistently higher MIC distributions across most antimicrobials. Among macrolides, azithromycin displayed identical MIC_50_ values in both collections (0.5 mg/L); however, the MIC_90_ increased from 0.75 mg/L in historic strains to 1.0 mg/L in contemporary isolates. A more pronounced temporal shift was observed for fluoroquinolones. For levofloxacin, MIC_50_ values remained unchanged (0.38 mg/L), whereas the MIC_90_ doubled from 0.5 mg/L in historic isolates to 1.0 mg/L in contemporary isolates. Ciprofloxacin exhibited an even more marked difference: although the MIC_50_ remained constant at 0.75 µg/mL, the MIC_90_ reached 1.0 µg/mL in both groups, but the cumulative inhibition curves revealed that a larger fraction of the contemporary isolates required concentrations closer to the upper bound of this range, indicating an overall reduced susceptibility (Table 3). In contrast, tigecycline susceptibility remained stable over time, with identical MIC_50_ and MIC_90_ values in both collections (1.5 mg/L and 2.0 mg/L, respectively). Similarly, erythromycin showed minimal temporal variation, with both historic and contemporary isolates displaying MIC_50_ and MIC_90_ values of 0.19 mg/L and 0.25 mg/L, respectively.

**Figure 3:**
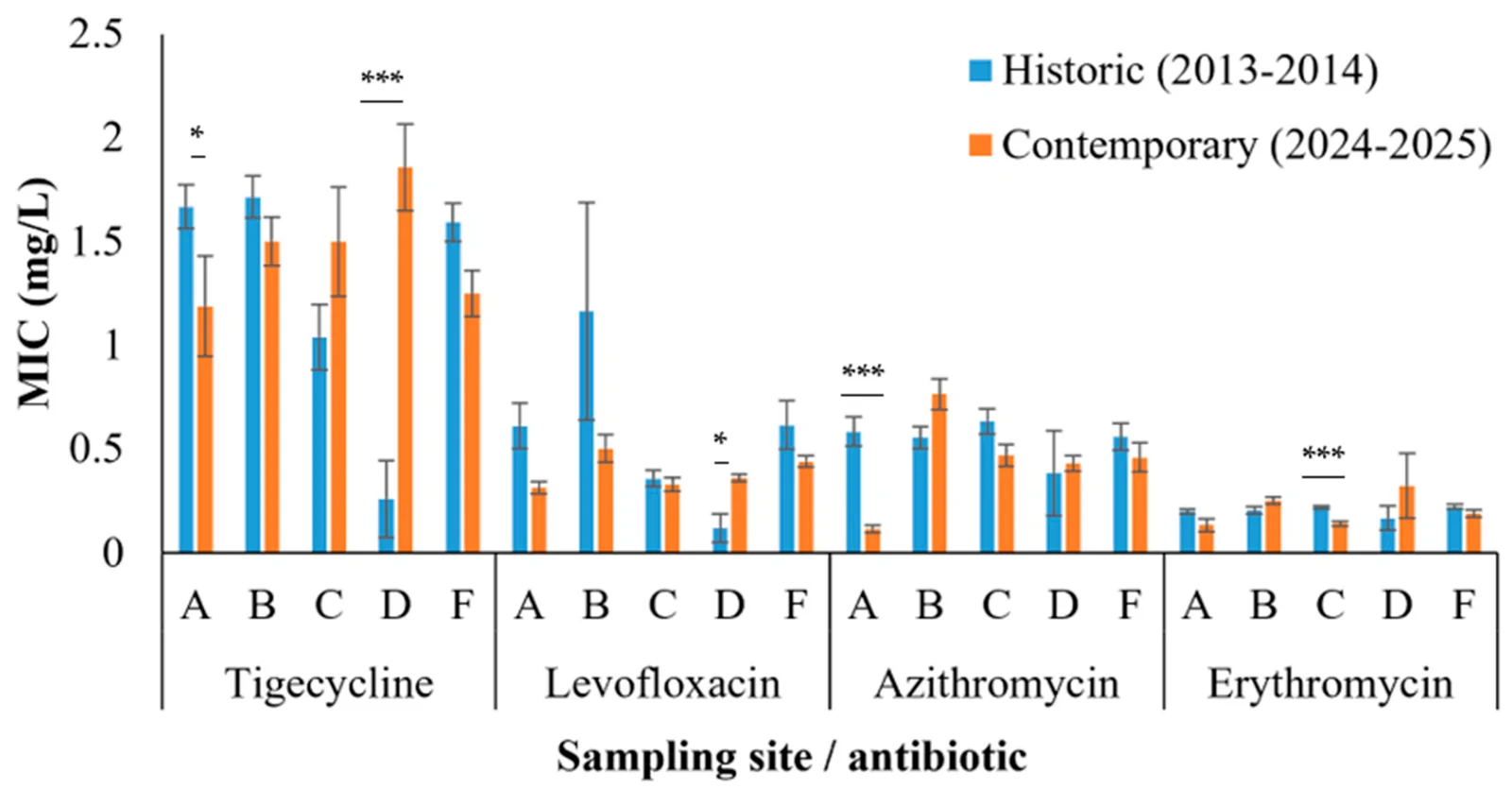
Mean minimum inhibitory concentrations (MICs, mg/L) of *L. pneumophila* isolates from five sampling points (A–F, point E was not included in this analysis as historic strains did not yield interpretable MIC values) for four antibiotics: tigecycline, levofloxacin, azithromycin, and erythromycin. Results are shown separately for isolates collected in 2013–2014 (historic; blue bars) and 2024–2025 (contemporary; orange bars). Error bars represent standard errors. Asterisks denote statistical significance levels as follows: * *p* < 0.05, and *** *p* < 0.001.

In addition, ANCOVA analyses were performed separately for each antibiotic. Each model included the sampling site and isolate age group (historic vs. contemporary) as fixed factors, with MIC values as the dependent variable. The permutation-based ANCOVA revealed statistically significant site-associated differences in MIC values for four of the six antibiotics tested: azithromycin (F_5,83_ = 9.43, *p* = 0.0001), erythromycin (F_5,83_ = 2.31, *p* = 0.0243), levofloxacin (F_5,83_ = 7.61, *p* = 0.0001), and tigecycline (F_5,83_ = 4.97, *p* = 0.0002). The magnitude and direction of these effects differed among antibiotics, with certain sites showing higher MICs in either historic or contemporary isolates (Figure 3).

Importantly, significant sampling site and isolate age interactions were detected for azithromycin (F_5,83_ = 10.27, *p* = 0.0001), levofloxacin (F_5,83_ = 5.73, *p* = 0.0003), tigecycline (F_5,83_ = 8.50, *p* = 0.0002), and erythromycin (F_5,83_ = 4.23, *p* = 0.0004), indicating that age-related susceptibility patterns differed between locations. Simple-effects Wilcoxon tests confirmed these site-specific trends: at point A, historic isolates had significantly higher azithromycin MICs (0.58 ± 0.07 mg/L) than contemporary isolates (0.12 ± 0.02 mg/L; *p* < 0.001). In contrast, at point D, contemporary isolates showed elevated MICs for erythromycin (0.17 ± 0.06 mg/L in historic vs. 0.32 ± 0.15 mg/L in contemporary; *p* = 0.0389), levofloxacin (0.12 ± 0.07 mg/L in historic vs. 0.36 ± 0.02 mg/L in contemporary; *p* = 0.048), and tigecycline (0.26 ± 0.19 mg/L in historic vs. 1.86 ± 0.21 mg/L in contemporary; *p* = 0.009), suggesting localized emergence of reduced susceptibility in recent strains. These increases were not only statistically significant but also quantitatively substantial. At point D, MIC values for erythromycin rose by 93.1%, for levofloxacin by 205.3%, and for tigecycline by as much as 615.4% in contemporary isolates compared with historic ones, highlighting a pronounced and localized shift toward reduced susceptibility (Figure 3).

To identify isolates with the broadest reduced-susceptibility profiles, a specific criterion was established: isolates were considered to exhibit broad-spectrum reduced susceptibility if their MIC values were at or above the 90th percentile for at least five of the six antibiotics tested. Two contemporary isolated strains were the only ones meeting this criterion, displaying MIC values at or above the 90th percentile for levofloxacin, tigecycline, ciprofloxacin, clarithromycin, and erythromycin, while azithromycin fell below the threshold for both isolates. This finding indicates a broad-spectrum reduced susceptibility phenotype spanning both macrolides and fluoroquinolones, potentially reflecting shared or co-selected resistance mechanisms.

## 4. Discussion

This study provides the first longitudinal comparison of *L. pneumophila* antimicrobial susceptibilities in the Middle East, using isolates collected from identical DWDS sites over a decade apart. By applying identical isolation and testing methodologies across both sampling campaigns, we demonstrate that antimicrobial susceptibility in *L. pneumophila* is neither static nor homogeneous but instead shaped by both temporal and spatial ecological factors. Several key findings emerge from this analysis.

### 4.1. Temporal Shifts in Susceptibility Patterns

Comparisons between historic (2013–2014) and contemporary (2024–2025) isolates revealed a consistent rightward shift in cumulative MIC distributions for most antibiotics. Macrolides, particularly azithromycin and clarithromycin, displayed stable MIC_50_ values (0.5 and 0.38 mg/L, respectively), yet azithromycin MIC_90_ increased from 0.75 mg/L in historic isolates to 1.0 mg/L in contemporary isolates. Notably, azithromycin MIC values in both historic and contemporary isolates in the present study were higher than those reported in European cohorts. Minetti et al. [14] reported azithromycin MIC_90_ values consistent with the EUCAST MIC distribution tables (MIC_90_ = 0.25 mg/L), while Gładysz et al. [39] described even lower MIC values in Polish isolates (MIC_50_ = 0.094 mg/L; MIC_90_ = 0.38 mg/L). Importantly, while historic isolates in our study already exhibited elevated MIC values relative to these European reports, contemporary isolates demonstrated a further shift toward higher MICs, placing the current isolate collection at the upper end of the reported azithromycin MIC distributions.

A similar pattern was observed for clarithromycin. MIC_90_ values in our study remained at 0.5 mg/L in both historic and contemporary isolate groups, which is higher than those reported by Minetti et al. [14] and Cruz et al. [40], where clarithromycin MIC_90_ values reached 0.25 mg/L and 0.125 mg/L, respectively. These findings suggest that clarithromycin susceptibility in the present isolate collection is shifted toward higher MIC values compared with other European reports, although without evidence of a pronounced temporal increase within the study period.

Fluoroquinolones exhibited more pronounced temporal changes. Levofloxacin MIC_90_ values doubled in our dataset, increasing from 0.5 mg/L in historic isolates to 1.0 mg/L in contemporary isolates, despite identical MIC_50_ values (0.38 mg/L). This shift is consistent with the findings of Minetti et al. [14] who reported a levofloxacin MIC_90_ of 1.0 mg/L. Ciprofloxacin susceptibility remained comparatively stable, with identical MIC_50_ (0.75 mg/L) and MIC_90_ (1.0 mg/L) values across both isolate groups; however, cumulative inhibition curves revealed a higher proportion of contemporary isolates clustering near the upper limit of the MIC distribution, indicative of reduced susceptibility. Overall, the MIC values observed in this study were higher than those reported by Cruz et al. [40] and Gładysz et al. [39], who documented ciprofloxacin MIC_90_ values of 0.25 mg/L and levofloxacin MIC_90_ values of 0.125 mg/L in Portuguese isolates, and MIC_90_ values of 0.38 mg/L for ciprofloxacin and 0.19 mg/L for levofloxacin in Polish isolates, respectively.

In contrast, tigecycline and erythromycin displayed largely stable MIC_50_ and MIC_90_ values over time. In our study, tigecycline MIC_50_ and MIC_90_ remained 1.5 and 2.0 mg/L, respectively, These values are lower than those reported by Minetti et al. [14], who observed substantially higher tigecycline MIC_90_ values (up to 16 mg/L) among Portuguese isolates, consistent with the upper range of the EUCAST wild-type MIC distribution. For erythromycin MIC_50_ and MIC_90_ values in both of our groups were 0.19 and 0.25 mg/L, respectively, in agreement with Minetti et al. [14], who reported erythromycin MIC_90_ values of up to 0.25 mg/L. By contrast, Gładysz et al. [39] and the EUCAST MIC distribution tables indicate a higher tentative upper wild-type MIC of 0.5 mg/L for erythromycin.

### 4.2. Spatial Heterogeneity Between Adjacent Sampling Sites

A striking outcome of this study is the detection of significant variation in MIC distributions between closely situated sampling sites within the same DWDS. For example, isolates from site A exhibited higher azithromycin MICs in the historic collection (mean = 0.58 mg/L) compared to contemporary isolates (0.12 mg/L), whereas isolates from site D in the contemporary collection showed elevated MICs for tigecycline (1.86 mg/L vs. 0.26 mg/L in historic isolates), levofloxacin (0.36 mg/L vs. 0.12 mg/L), and erythromycin (0.32 mg/L vs. 0.17 mg/L). These site-specific patterns reflect micro-scale environmental variation in selective pressures. Similar heterogeneity was noted in the systematic review by Pappa et al. [8], where MIC ranges for azithromycin varied from 0.064 to 4 mg/L across studies, underscoring that environmental context strongly influences resistance profiles. Future studies incorporating detailed plumbing characteristics and measurements of trace metals may help determine whether localized physicochemical conditions contribute to the site-specific susceptibility patterns observed in this study.

### 4.3. Emergence of Multi-Drug Reduced Susceptibility

By applying a stringent criterion (≥90th percentile MIC values across at least five of six antibiotics), we identified two contemporary isolates exhibiting broad-spectrum reduced susceptibility. These isolates demonstrated MIC values at or above 1 mg/L for levofloxacin, ciprofloxacin, clarithromycin, and erythromycin, while azithromycin remained below this threshold. While clinical breakpoints for *L. pneumophila* are lacking, these phenotypes are concerning.

To assess whether this pattern was reflected more broadly across the isolate collection, we examined stratified Spearman correlations among antibiotic MIC values. After FDR correction, contemporary isolates did not show a uniform strengthening of MIC correlations across all antibiotic classes. Instead, the correlation structure differed between sampling periods. Historic isolates exhibited broader macrolide-associated co-susceptibility patterns, including significant correlations between azithromycin–clarithromycin, azithromycin–erythromycin, and clarithromycin–erythromycin. In contrast, contemporary isolates showed a more restricted pattern, with significant correlations retained mainly between levofloxacin–ciprofloxacin, levofloxacin–clarithromycin, and azithromycin–erythromycin. Thus, although two contemporary isolates displayed a multi-drug reduced-susceptibility phenotype, the population-level data suggests a temporal restructuring of co-susceptibility patterns rather than a general increase in co-resistance.

Together, these findings point to a complex pattern of reduced susceptibility, rather than evidence for a single common resistance mechanism. Pereira et al. [12] detected antimicrobial resistance genes (ARGs), including efflux pump components such as *lpeAB*, in *L. pneumophila* from artificial water systems, linking genotypic data to phenotypic shifts. The presence of comparable resistance determinants in different geographical regions supports the notion that resistance evolution in *L. pneumophila* is a global process rooted in environmental reservoirs. However, the present study did not include molecular screening of the isolates exhibiting reduced susceptibility for antimicrobial resistance determinants. Future characterization of these isolates would provide valuable insight into the mechanisms underlying their reduced susceptibility.

### 4.4. Policy and Monitoring Implications

The results of this study support several recommendations for public health and water safety management. First, surveillance frameworks should incorporate longitudinal monitoring of antimicrobial susceptibility in *L. pneumophila* alongside routine quantification of bacterial loads, as recommended in the EUCAST guidance [41]. Second, susceptibility testing should be stratified by sampling site, recognizing that localized “hotspots” of reduced susceptibility may arise within a single DWDS. Third, standardized protocols for susceptibility testing and epidemiological cut-off determination should be adopted globally to harmonize datasets and enable robust comparisons across time and space.

Finally, the identification of two contemporary isolates with multi-drug reduced susceptibility, together with the observed temporal restructuring of MIC correlation patterns, supports targeted site-specific surveillance and biofilm management, rather than assuming a uniform system-wide emergence of co-resistance.

### 4.5. Limitation

This study has several limitations. First, the isolates originated from a single drinking water distribution system, which may limit the generalizability of the findings to other environmental settings. Second, antimicrobial susceptibility was assessed phenotypically, and molecular analyses to identify the genetic mechanisms underlying reduced susceptibility were not performed. Despite these limitations, the longitudinal design, identical sampling locations, and standardized methodology provide a robust framework for assessing temporal changes in antimicrobial susceptibility.

### 4.6. Future Directions

Future studies should expand environmental surveillance to additional drinking water distribution systems and geographical regions while integrating whole-genome sequencing and molecular characterization of isolates with reduced susceptibility. Combining phenotypic susceptibility testing with genomic analyses will improve our understanding of the mechanisms driving susceptibility shifts and support evidence-based strategies for environmental monitoring and risk management.

### 4.7. Conclusions

This decade-long comparative study demonstrates that *L. pneumophila* populations in environmental water systems undergo measurable shifts in antimicrobial susceptibility, with pronounced spatial heterogeneity, isolate-level multi-drug reduced susceptibility, and temporal restructuring of co-susceptibility patterns. When compared numerically with recent European studies, our findings place the studied isolates toward the upper end of reported MIC distributions for several antibiotics. Notably, MIC values for macrolides such as azithromycin and clarithromycin were higher in both historic and contemporary isolates than those reported in comparable European cohorts, with contemporary isolates exhibiting a further shift toward elevated MICs. These observations highlight emerging geographic and temporal variability in antimicrobial susceptibility patterns and underscore the urgent need for systematic environmental surveillance, harmonized susceptibility testing methodologies, and targeted policies addressing the environmental dimension of antimicrobial resistance in *L. pneumophila*.

## Figures and Tables

**Table 1:** Minimum inhibitory concentrations (MICs, mg/L) of the tested antimicrobial agents were determined for reference quality control strains used to validate antimicrobial susceptibility testing.

	*L. pneumophila* ATCC 33152	*Staphylococcus aureus* ATCC 29213	*Escherichia coli* ATCC 25922
Azithromycin	0.94	0.25	1
Levofloxacin	0.25	0.25	0.032
Tigecycline	1	0.25	0.19
Ciprofloxacin	0.5	0.25	0.012
Clarithromycin	0.19	0.19	32
Erythromycin	0.094	0.094	24

**Table 2:** MIC_50_, MIC_90_, and ECOFFs of six antibiotics (mg/L) tested against contemporary *L. pneumophila* isolates.

Antibiotic	N	MIC_50_	MIC_90_	MIC Range	ECOFF
Azithromycin	54	0.50	1.0	0.047–1.0	1.0
Levofloxacin	54	0.38	1.0	0.012–4.0	1.5
Tigecycline	54	1.5	2.0	0.016–2.0	2.0
Ciprofloxacin	53	0.75	1.0	0.016–1.5	2.0
Clarithromycin	53	0.38	0.50	0.016–0.75	1.0
Erythromycin	54	0.19	0.25	0.047–0.38	0.25

**Table 3:** Accumulated percentages (%) of *L. pneumophila* isolates inhibited at each tested concentration of the six antimicrobial agents. Values are presented as historic/contemporary, where “historic” corresponds to isolates collected in 2013–2014 and “contemporary” to isolates collected in 2024–2025. Percentages represent the cumulative fraction of isolates inhibited at or below each concentration. MIC_50_ and MIC_90_ values can be directly inferred from the table as the concentrations at which 50% and 90% of the isolates, respectively, were inhibited.

Conc. (mg/L)	0.012	0.016	0.032	0.047	0.064	0.094	0.125	0.19	0.25	0.38	0.5	0.75	1	1.25	1.5	2	3	4
Azithromycin				/2	2/6	8/	12/	15/	22/13	42/33	75/59	92/85	100/100					
Levofloxacin	/4		/6		/7	/13			20/19	72/65	98/81	/83	100/91		/96	/98		/100
Tigecycline		/4		/6		/11	/13	/15		2/		10/17	28/31		75/72	95/100	100/	
Ciprofloxacin		/4				/6		/8	2/9	5/	35/23	88/81	90/98		100/100			
Clarithromycin		/2			/4	/6	5/	10/13	38/21	72/68	100/94	/100						
Erythromycin				2/2		8/6	32/13	68/56	95/98	98/100				100/				

## Data Availability

The original contributions presented in this study are included in the article/Supplementary Material. Further inquiries can be directed to the corresponding author.

## Supplementary Materials

The following supporting information can be downloaded at https://www.mdpi.com/article/10.3390/pathogens15080852/s1; Table S1: Comparison of MIC50 and MIC90 values for historical Legionella pneumophila isolates. Comparison of MIC50 and MIC90 values reported by Sharaby et al. (2019) [24] and those obtained for historical isolates in the present study. The isolates analyzed here represent a subset of the original historical collection; therefore, differences in sample size and isolate composition should be considered when interpreting this comparison. Table S2: Stratified Spearman correlations among antibiotic MIC values. Values are Spearman’s rank correlation coefficients (r_s_), with Benjamini-Hochberg FDR-adjusted *p*-values in parentheses. n indicates the number of isolates with MIC values available for both antibiotics in each pair. FDR correction was applied separately within each correlation matrix. * pFDR < 0.05; ** pFDR < 0.01; *** pFDR < 0.001. Abbreviations: AZ, azithromycin; LE, levofloxacin; TGC, tigecycline; CI, ciprofloxacin; CH, clarithromycin; EM, erythromycin.
