# Supplementary material for: Longitudinal Antimicrobial Susceptibility Shifts in Environmental Legionella pneumophila from a Drinking Water Distribution System
Source: Pathogens. 2026 Aug 14;15(8):852. doi: 10.3390/pathogens15080852 (PMC13516451; doi:10.3390/pathogens15080852)
Supplement: Supplementary file 1 [file pathogens-15-00852-s001.zip › Supplementary Table S1.pdf]

**Supplementary Table S1.** Comparison of MIC50 and MIC90 values for historical *Legionella pneumophila* isolates. *Comparison of MIC50 and MIC90 values reported by Sharaby et al. (2019) and those obtained for historical isolates in the present study. The isolates analyzed here represent a subset of the original historical collection; therefore, differences in sample size and isolate composition should be considered when interpreting this comparison.*

| | **Sharaby et al. (2019) n = 105** | | **Present study n = 40** | |
|---|---|---|---|---|
| **Antibiotic** | **MIC50 (mg/L)** | **MIC90 (mg/L)** | **MIC50 (mg/L)** | **MIC90 (mg/L)** |
| **Azithromycin** | 0.38 | 0.75 | 0.50 | 0.75 |
| **Clarithromycin** | 0.064 | 0.25 | 0.38 | 0.50 |
| **Erythromycin** | 0.094 | 0.50 | 0.19 | 0.25 |
| **Levofloxacin** | 0.075 | 1.0 | 0.38 | 0.50 |
| **Ciprofloxacin** | 0.75 | 1.5 | 0.75 | 1.0 |
| **Tigecycline** | 0.50 | 1.5 | 1.5 | 2.0 |
