# Supplementary material for: Longitudinal Antimicrobial Susceptibility Shifts in Environmental Legionella pneumophila from a Drinking Water Distribution System
Source: Pathogens. 2026 Aug 14;15(8):852. doi: 10.3390/pathogens15080852 (PMC13516451; doi:10.3390/pathogens15080852)
Supplement: Supplementary file 1 [file pathogens-15-00852-s001.zip › Supplementary Table S2.pdf]

Supplementary Table S2. Stratified Spearman correlations among antibiotic MIC values.

Values are Spearman's rank correlation coefficients (r_s), with Benjamini-Hochberg FDR-adjusted p-values in parentheses. n indicates the number of isolates with MIC values available for both antibiotics in each pair.
FDR correction was applied separately within each correlation matrix. * pFDR < 0.05; ** pFDR < 0.01; *** pFDR < 0.001. Abbreviations: AZ, azithromycin; LE, levofloxacin; TGC, tigecycline; CI, ciprofloxacin; CH, clarithromycin; EM, erythromycin.

Antibiotic pair	All isolates	Historic isolates	Contemporary isolates
AZ-LE	0.34 (0.0029)**; n=93	0.33 (0.0705); n=40	0.32 (0.0724); n=53
AZ-TGC	0.08 (0.4698); n=93	0.36 (0.0456)*; n=40	−0.11 (0.5405); n=53
AZ-CI	0.16 (0.1705); n=92	0.12 (0.6194); n=40	0.15 (0.4173); n=52
AZ-CH	0.36 (0.0018)**; n=92	0.64 (<0.001)***; n=40	0.14 (0.4173); n=52
AZ-EM	0.57 (<0.001)***; n=94	0.58 (<0.001)***; n=40	0.51 (0.0014)**; n=54
LE-TGC	0.16 (0.1677); n=94	−0.05 (0.8300); n=40	0.28 (0.0949); n=54
LE-CI	0.39 (<0.001)***; n=92	0.24 (0.2135); n=40	0.47 (0.0025)**; n=52
LE-CH	0.44 (<0.001)***; n=92	0.44 (0.0179)*; n=40	0.47 (0.0025)**; n=52
LE-EM	0.22 (0.0619); n=93	0.41 (0.0257)*; n=40	0.07 (0.7048); n=53
TGC-CI	0.11 (0.3578); n=92	−0.03 (0.8716); n=40	0.16 (0.4173); n=52
TGC-CH	0.22 (0.0619); n=92	0.30 (0.1056); n=40	0.16 (0.4173); n=52
TGC-EM	0.18 (0.1392); n=93	0.37 (0.0455)*; n=40	0.06 (0.7297); n=53
CI-CH	0.22 (0.0619); n=93	0.10 (0.6621); n=40	0.30 (0.0836); n=53
CI-EM	−0.02 (0.8811); n=92	0.07 (0.7452); n=40	−0.15 (0.4173); n=52
CH-EM	0.25 (0.0357)*; n=92	0.49 (0.0071)**; n=40	0.04 (0.7738); n=52
